# The Diversity and Dynamics of Fungi in *Dryocosmus kuriphilus* Community

**DOI:** 10.3390/insects12050426

**Published:** 2021-05-10

**Authors:** Xiao-Hui Yang, Xiang-Mei Li, Dao-Hong Zhu, Yang Zeng, Lv-Quan Zhao

**Affiliations:** 1 Key Laboratory of Protein Chemistry and Developmental Biology of Fish of Education Ministry of China, State Key Laboratory of Developmental Biology of Freshwater Fish, College of Life Science, Hunan Normal University, Changsha 410081, China; xiangmeili@hunnu.edu.cn; 2 Laboratory of Insect Behavior and Evolutionary Ecology, Central South University of Forestry and Technology, Changsha 410004, China; dhzhu@csuft.edu.cn (D.-H.Z.); t20162281@csuft.edu.cn (Y.Z.); 3 Co-Innovation Center for Sustainable Forestry in Southern China, College of Forestry, Nanjing Forestry University, Nanjing 210037, China; zhaolvquan@njfu.edu.cn

**Keywords:** *Dryocosmus kuriphilus*, microbiome, diversity, fungal community, insect galls, high-throughput sequencing, *Castanea mollissima*

## Abstract

**Simple Summary:**

*Dryocosmus kuriphilus* is an invasive pest species which is native to China and is widely distributed in Asia, Europe and North America. *D. kuriphilus* induces insect galls on chestnut trees, and fungi can cause the necrosis of chestnut trees and the death of *D. kuriphilus*. The aim of this research was to investigate the potential role of *D. kuriphilus* in the transmission of fungi. We provide the first evidence that *D. kuriphilus* adults shared most fungal species with associated insect galls and the galled twigs of *Castanea mollissima*, and were dominated by *Botryosphaeria* sp., *Aspergillus* sp. and *Diaporthe* sp. Furthermore, we suggest that *D. kuriphilus* adults may be potential vectors of plant pathogens and mediate the transmission of fungi between chestnut trees.

**Abstract:**

*Dryocosmus kuriphilus* (Hymenoptera: Cynipidae) is a gall wasp that induces insect galls on chestnut trees and results in massive yield losses worldwide. Fungi can cause the necrosis of chestnut trees and the death of gall wasps. The aim of this research was to investigate the potential role of *D. kuriphilus* in the transmission of fungi. We sequenced the ribosomal RNA internal transcribed spacer region 1 of fungi in *D. kuriphilus* adults, associated insect galls and the galled twigs of *Castanea mollissima*, using high-throughput sequencing. We compared the species richness, α-diversity and community structure of fungi in *D. kuriphilus* adults, insect galls and the galled twigs. We provide the first evidence that *D. kuriphilus* adults shared most fungal species with associated insect galls and the galled twigs, and were dominated by *Botryosphaeria* sp., *Aspergillus* sp. and *Diaporthe* sp. We suggest *D. kuriphilus* adults may be potential vectors of plant pathogens and may facilitate the transmission of fungi between chestnut trees. Furthermore, the fungi may horizontally transmit among *D. kuriphilus* adults, associated insect galls and the galled twigs.

## 1. Introduction

Galling insects are highly specialized herbivores with the ability to induce the formation of insect galls on host plants [1,2]. Insect galls are the abnormal redifferentiation and growth of infested plant tissues, providing shelter and food for the galling insects [3,4]. The major groups of gall insects include gall wasps, gall midges, gall aphids, gall moths, psyllids and thrips [5].

*Dryocosmus kuriphilus* (Hymenoptera: Cynipidae) is a species of gall wasp that can result in massive reductions in the yields of different chestnut trees, including *Castanea henryi*, *Castanea mollissima* and *Castanea sativa* [6,7]. *D. kuriphilus* is one of the most successful invasive pests worldwide which is native to China and is widely distributed in Asia, Europe and North America [8,9]. The successful invasion and wide distribution of *D. kuriphilus* is associated with its parthenogenesis and life cycle [10]. The adults of *D. kuriphilus* lay eggs into the buds of host plants during the summer and their larvae overwinter inside the buds [11]. In the following spring, the *D. kuriphilus* larvae induce the formation of insect galls on host plants and feed until they achieve pupation [12]. Most nutrients in the insect galls are from the sites of photo-assimilate production or storage through phloem transport [13]. Thus, the occurrence of *D. kuriphilus* and their galls is shaped by the distribution of their host plant; their ecological interactions included oviposition, gall formation, parasitism and feeding.

Fungi interact with host plants, insect galls and galling insects in various ways [14]. Fungi can cause the necrosis of branches, leaves and fruits of many host plants [15]. Fungi act as pathogens, saprophytes or inquilines in insect galls [16]. The fungal pathogens could infect and destroy the insect gall tissue. For example, *Gnomoniopsis castaneae* was associated with the necrosis of insect galls of *D. kuriphilus* [17]. On the other hand, saprophytic fungi invade galls which are already dead or empty, whereas fungal inquilines live inside insect galls and feed upon gall tissues, but they do not directly parasitize the insect galls or the gall makers [18]. For the gall wasps, fungi may be one of the major causes of mortality [19]. For instance, some gall wasps suffered almost 100% mortality when their galls were artificially injected with spore suspensions of endophytic fungi *Discula quercina* [16]. Thus, fungi play an important role in the control of gall wasp [20].

Fungi infect many species of chestnut trees [21,22]. Previous studies have shown that the *D. kuriphilus*–induced insect galls were infected by *Colletotrichum acutatum*, *Cryphonectria parasitica* and *G. castaneae* [23]. The dominant fungi in the insect galls of *D. kuriphilus* vary across different regions. For example, the most abundant fungi in *D. kuriphilus*–induced galls in Italy were *Alternaria* spp., *G. castaneae* and *Trichothecium roseum* [24], whereas those in Spain were *C. acutatum*, *Fusarium* sp. and *G. castaneae* [25]. High-throughput sequencing analysis revealed that the fungal diversity within galls induced by *D. kuriphilus* was lower than that of their host plants [24]. Furthermore, *D. kuriphilus* has been reported to be infected by a range of fungi, including plant pathogens *C. parasitica* [26], *G. castaneae* [27,28], *Alternaria alternata*, *Botrytis* sp. and *Fusarium incarnatum* [29]. High-throughput sequencing analysis showed that the dominant fungi of *D. kuriphilus* in Italy included *C. acutatum*, *Epicoccum nigrum* and *Penicillium brevicompactum* [30].

In this current study, the richness, α-diversity and fungal community structure associated with *D. kuriphilus* adults, the insects’ galls and the galled twigs of *C. mollissima* in China were first compared at the species level, using high-throughput sequencing. We discuss the possibility of horizontal transmission of fungi and differences in the community structure of fungi in *D. kuriphilus* adults, associated insect galls and the galled twigs based on their ecological associations. In addition, we discussed the potential of *D. kuriphilus* adults acting as vectors of transmission of plant pathogens.

## 2. Materials and Methods

### 2.1. Sample Collection

*D. kuriphilus* adults, *D. kuriphilus*–induced insect galls and the galled twigs of *C. mollissima* were collected simultaneously from 20 trees at Huangqiao Town (27.02° N/110.85° E), China, in May 2018. All samples were snap-frozen for 30 min in liquid nitrogen after sampling. The frozen samples were kept in dry ice and transported to the laboratory at Hunan Normal University. Then the frozen samples were stored at −80 °C until processed. The *D. kuriphilus* adults were collected by removing them from insect galls with sterile scalpels and fine-pointed forceps to avoid potential contamination. The surfaces of *D. kuriphilus* adults, the insect galls and the galled twigs were washed with phosphate-buffered saline (PBS, pH = 7.4) buffer. The sample size of *D. kuriphilus* adults, associated insect galls and the galled twigs was nine for each group [30]. Each sample of *D. kuriphilus* adults included nine living individuals.

### 2.2. Total DNA Extraction and PCR Amplification

Total DNA from *D. kuriphilus* adults, associated insect galls and the galled twigs was extracted and purified with E.Z.N.A.^®^ soil DNA kit (Omega Bio-Tek, Norcross, GA, USA). Fungal ribosomal RNA internal transcribed spacer region 1 (ITS1) was amplified by using the primers ITS-F (5’-CTTGGTCATTTAGAGGAAGTAA-3’) and ITS-R (5’-GCTGCGTTCTTCATCGATGC-3’). The amplification was performed, using the GeneAmp PCR System 9700 (Applied Biosystems, London, UK) in a 20 μL reaction volume: 4 μL 5× TransStart FastPfu buffer, 2 μL dNTPs (2.5 mM each), 0.8 μL forward and reverse primer (5 μM), 0.4 μL Taq polymerase, 1 μL DNA template and 11 μL H_2_O. The PCR cycling conditions were 5 min at 95 °C, followed by 27 cycles of 30 s at 95 °C, 30 s at 53 °C, 45 s at 72 °C and a final elongation step of 10 min at 72 °C.

### 2.3. Library Construction and High-Throughput Sequencing

The PCR product was extracted from 2% agarose gel, following electrophoresis, and purified by using the AxyPrep DNA Gel Extraction Kit (Axygen Biosciences, Union City, CA, USA). The PCR product was quantified by using a Quantus™ Fluorometer (Promega, Madison, Wisconsin, WI, USA). The libraries were prepared by using NEXTFLEX Rapid DNA-Seq Kit (Bioo Scientific, Austin, TX, USA), and high-throughput paired-end sequencing was performed on the Illumina MiSeq (PE300) sequencing platform (Illumina, San Diego, CA, USA). Library preparation and sequencing were carried out by Majorbio Bio-Pharm Technology Co. Ltd. (Shanghai, China). The raw data were deposited into the NCBI Sequence Read Archive (SRA) database under Accession Number PRJNA725226.

### 2.4. Bioinformatics Analysis

The raw ITS1 gene sequencing reads were quality-filtered by fastp software [31] and merged, using FLASH software [32]. The sequences fulfilling the following criteria were used for the subsequent analysis: sequence length >200 bp, no ambiguous bases and mean quality score ≥20. After quality filtering, high-quality reads were clustered into Operational Taxonomic Units (OTU) at a similarity cutoff value of 97%, using UPARSE, and were screened for chimeras, using USEARCH version 7.1 [33]. The chimeric sequences were identified and then removed. The taxonomy of each OTU representative sequence was analyzed and annotated from the phylum to species level by the Ribosomal Database Project (RDP) classifier version 2.4 [34] and the UNITE database for molecular identification of fungi, using a confidence threshold of 0.7. For each sample, 54,497 sequences were randomly selected to generate an OTU table. The 54,497 represents the sequence count of the sample with the smallest acceptable number of sequences. The OTU table, which recorded the abundance and taxonomy of each OTU, was used for the subsequent statistical analysis.

### 2.5. Statistical Analysis

Statistical analysis was performed by using R version 3.6.3 (https://www.r-project.org, 26 February 2020). We counted the number of unique and common fungi in *D. kuriphilus* adults, associated insect galls and the galled twigs of *C. mollissima* at the species level.

The Sobs index and the Shannon index measures were used to evaluate the observed species richness and α-diversity, respectively, of the fungal community of *D. kuriphilus* adults, associated insect galls and the galled twigs at the species level. Sobs index refers to the total number of fungal species observed in *D. kuriphilus* adults, associated insect galls and the galled twigs. Data relating to Sobs and Shannon index were tested for normal distribution (Shapiro–Wilk test) and homogeneity of variance (Bartlett’s test). The data of the Sobs index were approximately normally distributed, and the variance was homogeneous across groups. Thus, one-way analysis of variance (ANOVA) was used to evaluate whether there were overall significant differences among the Sobs index measures of different groups; if significant, the Tukey–Kramer test was then used to carry out multiple pairwise comparisons of the groups. The variance of the Shannon index was not homogeneous across groups, so the Kruskal–Wallis nonparametric test was used to evaluate whether there were overall significant differences among the Shannon index of different groups, with the Dunn test being used for multiple comparisons if the Kruskal–Wallis test was significant.

Principal coordinate analyses (PCoA) were performed to compare the fungal community structure of *D. kuriphilus*, associated insect galls and the galled twigs. First, the overall difference in community structure was assessed, using permutational multivariate analysis of variance (PERMANOVA). PERMANOVA was carried out by using the “adonis” function in the “vegan” package in R based on the weighted UniFrac distance with 1000 permutations [35]. Second, PCoA was carried out based on weighted UniFrac distance, using the “pcoa” function in the R package “ape” [36].

The linear discriminant analysis (LDA) Effect Size (LEfSe) (http://huttenhower.sph.harvard.edu/galaxy/, 17 March 2021) was used to reveal predominant fungi in *D. kuriphilus* adults, associated insect galls and the galled twigs. The Kruskal–Wallis test was used to detect those fungal taxa where the relative abundance was significantly different among *D. kuriphilus* adults, associated insect galls and the galled twigs from the phylum to the species level. Then, the linear discriminant analysis (LDA) was used to calculate the effect size of each taxon; the higher the LDA score, the greater the influence of taxa on the difference. For fungi with an LDA score greater than 4, the relative abundance of the fungi was showed in bubble chart and the Dunn test was used for multiple pairwise comparisons. The predominant fungi refer to the fungi with an LDA score greater than 4 and the highest abundances among *D. kuriphilus* adults, associated insect galls and the galled twigs.

## 3. Results

### 3.1. The Fungal Community Composition of D. kuriphilus Adults, Associated Insect Galls and the Galled Twigs of C. mollissima

There was a total of four phyla, 22 classes, 56 orders, 116 families, 117 genera, 248 species and 385 OTUs in the fungal community of *D. kuriphilus* adults, associated insect galls and the galled twigs (Table 1). Insect galls had the most fungi, followed by the galled twigs, with *D. kuriphilus* having the fewest fungi from the phylum to species level (Table 1). The fungal communities of *D. kuriphilus* adults, associated insect galls and the galled twigs had 176, 241 and 221 species, respectively (Table 1). At the phylum and class level, the taxa with the highest abundances were identical in the fungal communities of *D. kuriphilus* adults, associated insect galls and the galled twigs (Table 2). At the order, family, genus, species and OTU levels, the taxa with the highest abundances in *D. kuriphilus* adults were not the same as that in the insect galls and the galled twigs, whereas the taxa with the highest abundances were identical in the fungal community of the insect galls and the galled twigs (Table 2). The fungi with the highest abundances of *D. kuriphilus* adults were *Botryosphaeria* sp. (Table 2).

### 3.2. The Unique and Common Fungi of D. kuriphilus Adults, Associated Insect Galls and the Galled Twigs of C. mollissima

**Figure 1:**
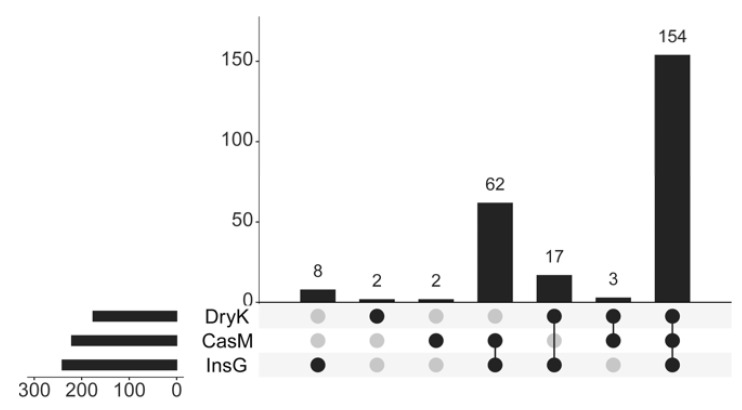
The number of unique and common fungal species in DryK, InsG and CasM at the species level. Dryk, InsG and CasM represent *Dryocosmus kuriphilus* adults, associated insect galls and the galled twigs of *Castanea mollissima*, respectively. The black point indicates the fungi in DryK, InsG and CasM at the species level. The gray point indicates the fungi were not in DryK, InsG and CasM at the species level. The horizontal axis at the lower left shows the total number of fungal species in DryK, InsG and CasM. The vertical axis shows the number of fungi unique or common to DryK, InsG and CasM.

A total of 154 fungi were common to *D. kuriphilus* adults, associated insect galls and the galled twigs (Figure 1). The relative abundance of the fungi common to *D. kuriphilus* adults, associated insect galls and the galled twigs was 99.36%, 98.03% and 98.71%, respectively (Table 3). The numbers of unique fungi in *D. kuriphilus* adults, associated insect galls and the galled twigs were eight, two and two, respectively (Figure 1). The relative abundance of unique fungi in *D. kuriphilus* adults, associated insect galls and the galled twigs was 0.14%, 0.03% and 0.04%, respectively (Table 3).

### 3.3. The Richness and α-Diversity at the Species Level of the Fungal Communities of D. kuriphilus Adults, Associated Insect Galls and the Galled Twigs of C. mollissima

**Figure 2:**
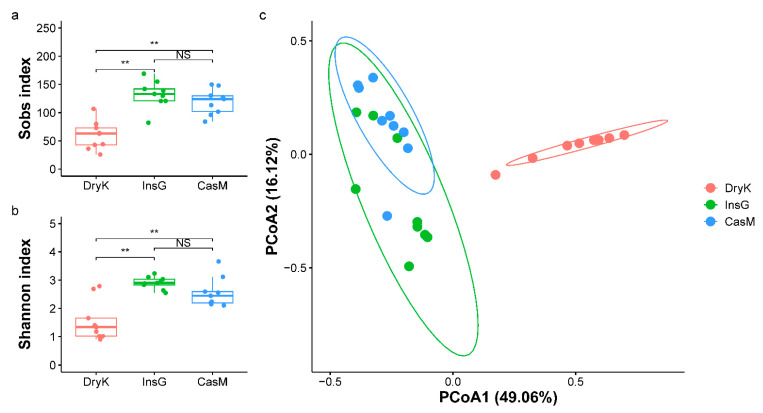
The fungal richness, α-diversity and community structure of DryK, InsG and CasM at the species level. (**a**). Boxplot of fungal richness at the species level of DryK, InsG and CasM, as measured by the Sobs index. (**b**). Boxplot of fungal α-diversity at the species level of DryK, InsG and CasM, as measured by the Shannon index. ** Indicates a significant difference (*p* < 0.01) (ANOVA in (**a**); Kruskal–Wallis in (**b**)), and NS indicates that any difference is not significant. The top and bottom horizontal lines of the boxplot indicate 25th and 75th percentiles, respectively. The lines within the box indicate median values, while vertical lines indicate the 10th and 90th percentiles. (**c**). Principal coordinates analysis (PCoA) based on weighted UniFrac distance at the species level of the fungal community structure of DryK, InsG and CasM. The horizontal and vertical axes indicate the first and second principal coordinates (PCoA1 and PCoA2, respectively). The percentage indicates the proportion of the total variation explained by each principal coordinate. The ellipses represent the 95% confidence interval around the centroid for DryK, InsG or CasM. DryK, InsG and CasM represent *Dryocosmus kuriphilus* adults, associated insect galls and the galled twigs of *Castanea mollissima*, respectively.

The observed species richness (ANOVA, F_2,24_ = 23.36, *p* < 0.01) and α-diversity (Kruskal–Wallis test, H_2,24_ = 12.98, *p* < 0.01) measures at the species level differed significantly among the fungal communities of *D. kuriphilus* adults, associated insect galls and the galled twigs (Figure 2). The observed species richness (Tukey–Kramer’s test, *p* < 0.01) and α-diversity measures (Dunn test, *p* < 0.01) of the fungal community of *D. kuriphilus* adults were significantly lower than those of associated insect galls and the galled twigs at the species level (Figure 2), whereas the species richness (Tukey–Kramer’s test, *p* = 0.27) and α-diversity (Dunn test, *p* = 0.115) of the fungal communities were not significantly different between associated insect galls and the galled twigs at the species level (Figure 2). Furthermore, there was an overall significant difference among the fungal community structures of *D. kuriphilus* adults, associated insect galls and the galled twigs (PERMANOVA, R^2^ = 0.53, *p* < 0.01) (Figure 2). PCoA analysis indicated that the fungal community structure in *D. kuriphilus* adults was clearly different from that of associated insect galls and the galled twigs (Figure 2).

### 3.4. The Predominant Fungal Species of D. kuriphilus Adults, Associated Insect Galls and the Galled Twigs of C. mollissima

**Figure 3:**
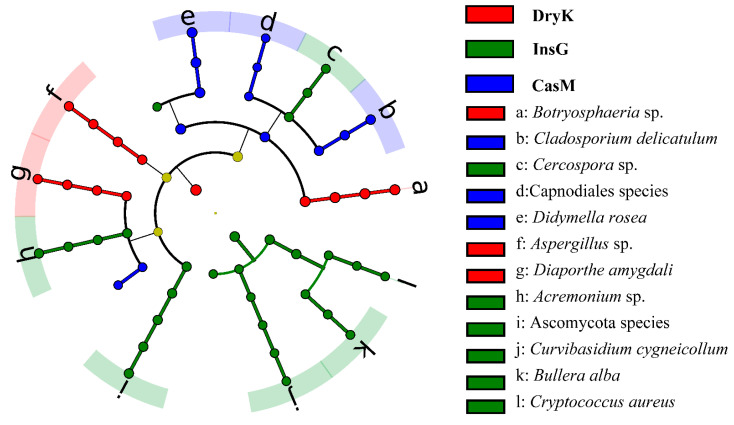
The LEfSe plot of the predominant fungi in DryK, InsG and CasM. The LEfSe represents the linear discriminant analysis effect size. DryK, InsG and CasM represent *Dryocosmus kuriphilus* adults, associated insect galls and the galled twigs of *Castanea mollissima*, respectively. The cladogram levels, from the inner to outer rings, stand for phylum, class, order, family, genus and species. The red, green and blue nodes of the cladogram show the predominant fungi in the DryK, InsG and CasM from the phylum to species level, respectively. The yellow nodes show the non-dominant fungi in DryK, InsG and CasM. The letters from a to l show the scientific name of predominant fungal species in the DryK, InsG and CasM. The Ascomycota species indicates unclassified species of the Ascomycota phylum. The Capnodiales species indicates unclassified species of the Capnodiales order.

The LEfSe analysis showed that a total of two phyla, four classes, ten orders, 14 families, 12 genera and 12 species were predominant in the fungal communities of *D. kuriphilus* adults, associated insect galls and the galled twigs (Figure 3). The fungal community of *D. kuriphilus* adults was dominated by one phylum, three orders, three families, three genera and three species. The fungal community of associated insect galls was dominated by one phylum, four classes, four orders, seven families, six genera and six species, whereas the fungal community of the galled twigs was dominated by three orders, four families, three genera and three species (Figure 3).

**Figure 4:**
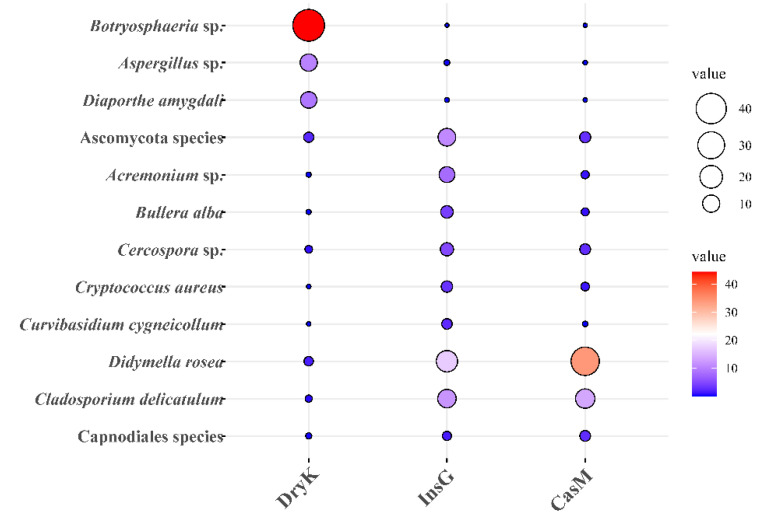
The bubble chart of relative abundance of predominant fungi in the DryK, InsG and CasM at the species level. DryK, InsG and CasM represent *Dryocosmus kuriphilus*, associated insect galls and the galled twigs of *Castanea mollissima*, respectively. The area and color of circle show the relative abundance of predominant fungi in the DryK, InsG and CasM. The relative abundance is expressed as the percentage of predominant fungi in the total fungi. The Ascomycota species indicates unclassified species of the Ascomycota phylum. The Capnodiales species indicates unclassified species of the Capnodiales order.

Notably, it was shown for the first time that *Botryosphaeria* sp., *Aspergillus* sp. and *Diaporthe* sp. were predominant in the fungal community of *D. kuriphilus* adults (Figure 3, Table 4). The relative abundances of *Botryosphaeria* sp., *Aspergillus* sp. and *Diaporthe* sp. in *D. kuriphilus* adults were 44.27%, 10.07% and 8.91%, respectively (Figure 4 and Figure S1). Furthermore, the insect galls were dominated by six fungi, namely Ascomycota species, Acremonium sp., *Bullera alba*, *Cercospora* sp., *Cryptococcus aureus* and *Curvibasidium cygneicollum* (Figure 3, Table 4), whereas the galled twigs were dominated by three fungi, namely *Didymella rosea*, *Cladosporium delicatulum* and Capnodiales species (Figure 3 and Table 4).

## 4. Discussion

### 4.1. The Possibility of Fungal Horizontal Transmission among D. kuriphilus Adults, Associated Insect Galls and the Galled Twigs of C. mollissima

To our knowledge, this study provided the first evidence that *D. kuriphilus* adults, their associated insect galls and the galled twigs share most of the species in the fungal community. Previous studies have shown that the insect galls of *D. kuriphilus* and host plants shared *C. aureus* [24], *C. cygneicollum* [37], *Cercospora* spp. [38], *Cladosporium* spp. [21] and *D. rosea* [25,39]. Furthermore, *D. kuriphilus* and associated insect galls shared *C. parasitica* [26], *Fusarium* spp. [29] and *G. castaneae* [27]. Therefore, the sharing of fungi among *D. kuriphilus* adults, associated insect galls and the galled twigs may be common.

We speculated that the fungi might horizontally transmit among *D. kuriphilus* adults, associated insect galls and the galled twigs. We suggest that structural (vascular) connections, transport of substances, contact and feeding relationships play an essential role in the potential horizontal transmission among *D. kuriphilus* adults, associated insect galls and the galled twigs.

The insect galls of *D. kuriphilus* are structurally connected with the galled twigs [40]. This structural connection provides a physical route for the horizontal transmission of fungi between *D. kuriphilus*–induced insect galls and the galled twigs. For example, endophytic fungi can grow into insect galls from the neighboring leaf in the form of mycelia or by directly penetration of the gall via spores [16]. Moreover, the supply of water and most nutrients to insect galls are obtained from the host plants via xylem vessels and phloem sieve tubes, respectively [41,42]. The spores of *Ceratocystis fagacearum* could spread from the primary infection site to other parts of the host plant through the xylem vessels and the phloem sieve tubes [43,44]. Thus, the transport of water and nutrients may provide favorable conditions for the horizontal transmission of fungi between *D. kuriphilus*–induced galls and the galled twigs. Furthermore, *D. kuriphilus* lives in the gall chambers of insect galls, making constant contact with the insect galls before eclosion [45]. During this contact process, the fungi associated with the insect galls may adhere to the exoskeleton surface of *D. kuriphilus* adults or be collected and transported within the body of *D. kuriphilus*. The fungi associated with insect galls may enter the digestive system of *D. kuriphilus* when the latter feeds on the insect galls. Therefore, such contact and feeding relationships are conducive to the horizontal transmission of fungi between *D. kuriphilus* adults and the insect galls.

### 4.2. The Differences in Fungal Community Structure among D. kuriphilus Adults, Associated Insect Galls and the Galled Twigs of C. mollissima

The differences in fungal community structure between the insect galls and the galled twigs may be associated with the differences in chemical composition and content between the insect galls and the galled twigs. Previous studies have confirmed differences in chemical composition and concentration between the host plant and both aphid galls [46] and midge galls [47]. The fungal community structures of aphid galls [48] and midge galls [49] also differed from those associated with the corresponding host plants. For the insect galls of *D. kuriphilus* and other gall wasps, the chemical composition, as well as the concentrations of amino acids, carbohydrates, lipids, lignin and secondary metabolites were markedly different from those of the host plants [42,50,51,52,53,54]. We propose that the chemical components and concentrations of insect galls induced by *D. kuriphilus* may affect the fungal community structure and provide a particular habitat for the fungi associated with the insect galls. For example, Cornell has shown that the high tannin concentration in the insect galls of galls wasps prevents the colonization of some fungi [55].

Furthermore, the ability of fungi to utilize particular plant chemicals may also associate with the differences in fungal community structure between the insect galls and the galled twigs. The lignification degree of cynipid galls is higher than that of the host plants [56]. Lignin is a complex, polyphenolic macromolecule, which is refractory to degradation and assimilation [57]. However, some fungi, such as the white-rot fungi, can break down and use lignin by producing diverse extracellular oxidases, including phenol oxidases, lignin peroxidase and manganese peroxidase [58]. These fungi, which can utilize the substances making up the insect galls of *D. kuriphilus*, may be better adapted to the environment of insect galls.

The fungal community structure of *D. kuriphilus* adults was obviously different from that of insect galls. We suggest that the inter-kingdom barriers between *D. kuriphilus* and insect galls may prevent the colonization of some fungi and hence contribute to fungal community structure differences between *D. kuriphilus* adults and associated insect galls. Fungal colonization at the cross-kingdom level is not as well-known as that within the animal or plant kingdoms. The fungi must come into close and frequent contact with potential hosts and overcome the host defense of another kingdom [59,60].

### 4.3. D. kuriphilus Adults as Potential Vectors of PLANT Pathogens

Many species of the *Botryosphaeria*, *Aspergillus* and *Diaporthe* genera are plant pathogens [61,62,63]. Previous studies had shown that *C. mollissima* is attacked by a range of fungi, including plant pathogens such as *Botryosphaeria dothidea* [63], *Aspergillus* sp. [64,65] and *Diaporthe nobilis* [66]. Furthermore, many fungi have been isolated from the insect galls induced by *D. kuriphilus*, including *B. dothidea* [24,67,68], *D. nobilis* [24] and *Aspergillus* spp. [28]. We noticed that plant pathogens *B. dothidea* was isolated from five phytophagous insects and the dispersal and propagule pressure of *Botryosphaeria* spp. in oak trees were affected by insect vectors [69]. Thus, the predominant fungi in *D. kuriphilus* adults, such as *Botryosphaeria* sp., *Aspergillus* sp. and *Diaporthe* sp., may be plant pathogens.

The ovipositor of *D. kuriphilus* is a needle-like apparatus used to introduce wasp eggs into buds of the host plants and may result in fresh wounds in buds [30,49]. The injuries provide entry points for fungi and a potential approach for the fungal transmission between *D. kuriphilus* and the host tree, *C. mollissima*. For example, spores of the parasitic *C. parasitica* infected host plants through fresh wounds [27,67]. Panzavolta et al. indicated that the galling insects associated with the transport of plant pathogens to oak trees [70]. Here, we suggest that *D. kuriphilus* adults may be potential vectors of plant pathogens and can mediate the transmission of fungi between chestnut trees, and the pathogen pervasiveness of chestnut trees may be enhanced by their association with *D. kuriphilus*.

### 4.4. The Predominant Fungi in D. kuriphilus

The fungi associated with galling insects can be saprotrophs, symbionts and insect pathogens [71]. The available literature suggests that the death of gall wasps was associated with several fungi, including *Cladosporium* sp. [72], *D. quercina* [16], *G. castaneae* [28], *Gnomoniopsis smithogilvyi*, *Fusarium oxysporum* and *Fusarium avenaceum* [73]. Previous studies have confirmed that some species of the *Aspergillus* genus contribute to the death of members of the insect orders Hymenoptera [74], Lepidoptera [75], Coleoptera [76] and Diptera [77]. Furthermore, some species of the *Diaporthe* genus are pathogens of dipteran [78] and lepidopteran species [20,79] and can result in the death of insects [61]. However, there is no firm evidence indicating that *Botryosphaeria* sp., *Aspergillus* sp. or *Diaporthe* sp. can result in the death of *D. kuriphilus* adults.

In future studies, we will focus on the isolation and cultivation of predominant fungi in *D. kuriphilus* adults and plan to evaluate the role of these fungi.

## 5. Conclusions

In conclusion, this study indicated that *D. kuriphilus* adults, associated insect galls and the galled twigs of *C. mollissima* shared most of the species in the fungal community for the first time. This study also provided the first evidence that *Botryosphaeria* sp., *Aspergillus* sp. and *Diaporthe* sp. were predominant in the fungal community of *D. kuriphilus*.

We suggest that structural (vascular) connections, the transport of substances, contact, feeding and oviposition relationships play an important role in the potential horizontal transmission of fungal species among *D. kuriphilus* adults, the associated insect galls and the galled twigs. Furthermore, differences in fungal community structure among *D. kuriphilus* adults, the insect galls and the galled twigs may be associated with differences in the chemical composition and concentrations between insect galls and galled twigs, differences in the ability of fungi to use key chemicals and cross-kingdom barriers between *D. kuriphilus* and the plant tissue forming the insect galls. In addition, *Botryosphaeria* sp., *Aspergillus* sp. and *Diaporthe* sp. may be plant pathogens. We suggest that *D. kuriphilus* adults may be potential vectors of plant pathogens.

## Figures and Tables

**Table 1:** The total number of fungi in *Dryocosmus kuriphilus* adults, associated insect galls and the galled twigs of *Castanea mollissima* at different taxon levels.

	Phylum	Class	Order	Family	Genus	Species	OTU
*D. kuriphilus* adults	4	20	46	93	133	176	250
Insect galls	4	22	56	114	171	241	375
Galled twigs	4	21	53	107	164	221	347
Total	4	22	56	116	177	248	385

OTU: operational taxonomic unit.

**Table 2:** The fungi with the highest relative abundance in *Dryocosmus kuriphilus* adults, associated insect galls and the galled twigs of *Castanea mollissima* at different taxon levels.

	Phylum	Class	Order	Family	Genus	Species	OTU
*D. Kuriphilus* adults	Ascomycota (97.28) ^A^	Dothideomycetes (51.79) ^A^	Botryosphaeriales (44.91) ^A^	Botryosphaeriaceae (44.91) ^A^	*Botryosphaeria* (44.90) ^A^	*Botryosphaeria* sp. (44.90) ^A^	OTU726 (44.90) ^A^
Insect galls	Ascomycota (79.26) ^A^	Dothideomycetes (49.60) ^A^	Pleosporales (25.55) ^A^	Didymellaceae (18.36) ^A^	*Didymella* (17.92) ^A^	*Didymella rosea* (17.89) ^A^	OTU1183 (17.89) ^A^
Galled twigs	Ascomycota (88.43) ^A^	Dothideomycetes (66.33) ^A^	Pleosporales (41.69) ^A^	Didymellaceae (35.72) ^A^	*Didymella* (34.84) ^A^	*D. rosea* (34.82) ^A^	OTU1183 (34.82) ^A^

^A^ The numbers inside the parentheses represent the relative abundance expressed as the percentage of this taxon abundance in the fungal community of *D. kuriphilus* adults, associated insect galls or the galled twigs.

**Table 3:** The percentage of fungi unique and common to *Dryocosmus kuriphilus* adults, associated insect galls and the galled twigs of *Castanea mollissima*.

Source	Unique Fungi	Fungi Common to DryK and InsG	Fungi Common to DryK and CasM	Fungi Common to InsG and CasM	Fungi Common to DryK, InsG and CasM
*D. Kuriphilus* adults	0.14 ^A^	0.42 ^A^	0.08 ^A^	-	99.36 ^A^
Insect galls	0.03 ^A^	0.40 ^A^	-	1.54 ^A^	98.03 ^A^
Galled twigs	0.04 ^A^	-	0.06 ^A^	1.20 ^A^	98.71 ^A^

^A^ The numbers indicate the relative abundance expressed as a percentage of this taxon abundance based on the number of reads in the fungal community of *D. kuriphilus* adults, associated insect galls or the galled twigs.

**Table 4:** The predominant fungi in *Dryocosmus kuriphilus* adults, associated insect galls and the galled twigs of *Castanea mollissima*.

Predominant Fungi	Reported in *D.* *kuriphilus*	Reported in Insect Galls	Reported in Galled Twigs
*D. kuriphilus* group			
*Botryosphaeria* sp.	No	Fernandez-Conradi et al., 2019	Fernandez-Conradi et al., 2019.
*Aspergillus* sp.	No	Vannini et al., 2017.	Overy et al., 2003; Donis-González et al., 2016
*Diaporthe* sp.	No	Fernandez-Conradi et al., 2019.	Zhang et al., 2018; Fernandez-Conradi et al., 2019.
Insect galls group			
Ascomycota species	–	–	–
*Acremonium* sp.	No	No	Driss, 2019
*Bullera alba*	No	No	Driss, 2019
*Cercospora* sp.	No	Vinale et al., 2014.	Vinale et al., 2014.
*Cryptococcus aureus*	No	Fernandez-Conradi et al., 2019.	LaBonte et al., 2018; Fernandez-Conradi et al., 2019.
*Curvibasidium cygneicollum*	No	Driss, 2019.	Driss, 2019.
Galled twigs group			
*Didymella rosea*	No	No	LaBonte et al., 2018.
*Cladosporium delicatulum*	Fernandez-Conradi et al., 2019	Seddaiu et al., 2017.	Zhang et al., 2009; LaBonte et al., 2018
Capnodiales species	–	–	–

The Ascomycota species indicates unclassified species of the Ascomycota phylum. The Capnodiales species indicates unclassified species of the Capnodiales order.

## Data Availability

The raw data were deposited into the NCBI Sequence Read Archive (SRA) database under Accession Number PRJNA725226.

## Supplementary Materials

The following are available online at https://www.mdpi.com/article/10.3390/insects12050426/s1. Figure S1: The relative abundance of fungi predominant in the *Dryocosmus kuriphilus* adults, associated insect galls and the twigs of *Castanea mollissima* at the species level.
