# Supplementary material for: The Diversity and Dynamics of Fungi in Dryocosmus kuriphilus Community
Source: Insects. 2021 May 10;12(5):426. doi: 10.3390/insects12050426 (PMC8151921; doi:10.3390/insects12050426)
Supplement: Supplementary file 1 [file insects-12-00426-s001.zip › Supplementary caption.pdf]

Figure S1. The relative abundance of fungi enriched in the *Dryocosmus kuriphilus* adults, associated insect galls and the twigs of *Castanea mollissima* at the species level. DryK, InsG and CasM represent *D. kuriphilus* adults, associated insect galls and the galled twigs of *C. mollissima*, respectively. *, ** and*** indicate significant difference (* is for $p<0.05$, ** is for $p<0.01$ and *** for $p<0.001$) and the NS indicates that any differences are not significant. The relative abundance is expressed as the percentage of enriched fungi in the total fungi.
