# Supplementary figures and images for: The Diversity and Dynamics of Fungi in Dryocosmus kuriphilus Community
Source: Insects. 2021 May 10;12(5):426. doi: 10.3390/insects12050426 (PMC8151921; doi:10.3390/insects12050426)

Group ■ DryK ■ InsG ■ CasM

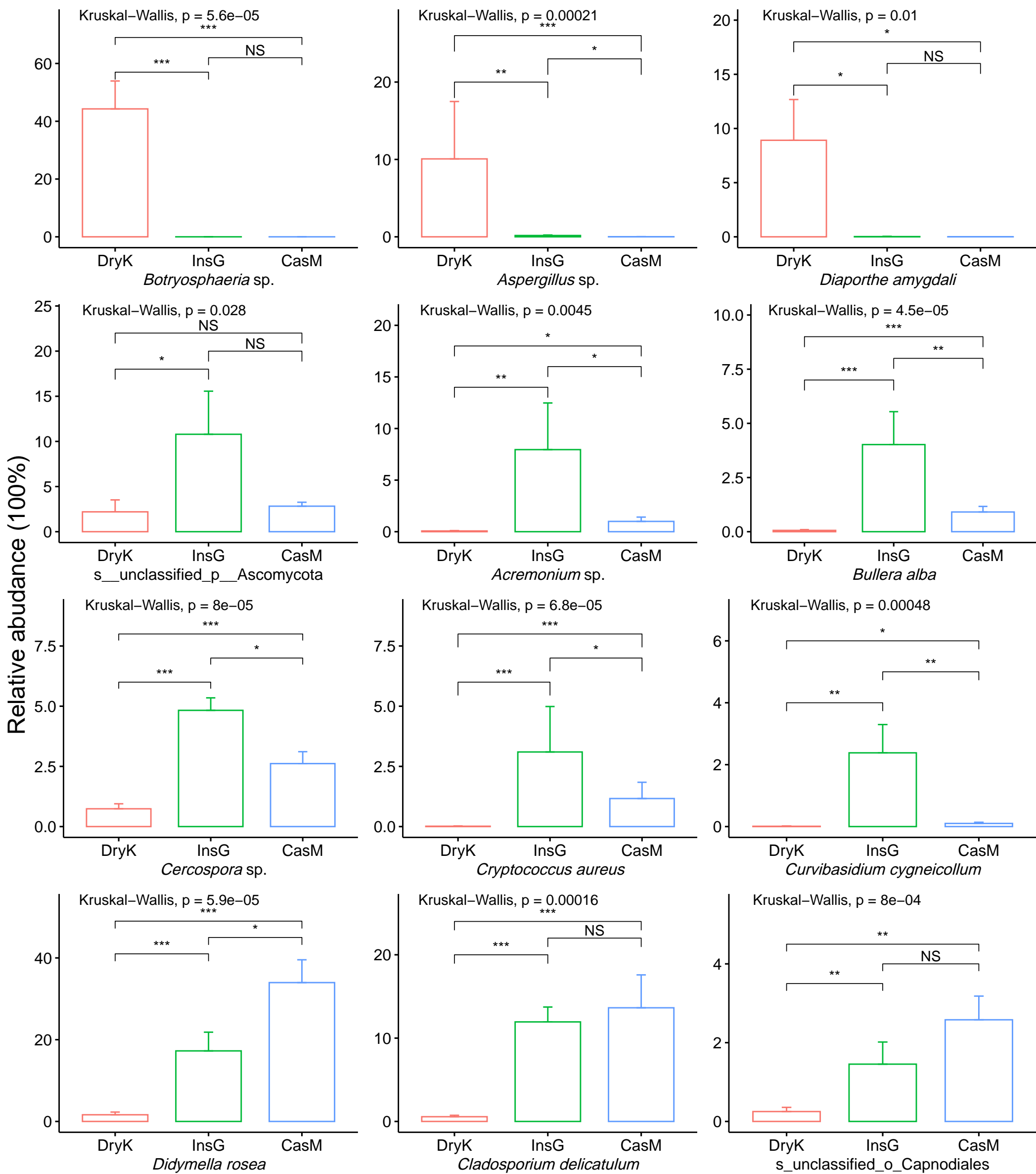

Supplement: Supplementary file 1 [file insects-12-00426-s001.zip › Figure S1.pdf]
